# Norcantharidin Ameliorates Experimental Ulcerative Colitis Through Epigenetic and Metabolic Reprogramming Involving IL-6/DNMT1/SOCS3 and AMPK/SIRT1/FOXO3a-Nrf2 Signaling

**DOI:** 10.3390/ijms27156666

**Published:** 2026-07-26

**Authors:** Eman H. Yousef, Samia S. Hawas, Mohamed M. Salama, Mostafa E. Metawee, Hader I. Sakr, Sumaiah J. Alarfaj, Amany A. Alzokaky

**Affiliations:** 1 Department of Pharmacology and Biochemistry (Biochemistry), Faculty of Pharmacy, Horus University-Egypt, New Damietta 34518, Egypt; 2 Department of Pharmaceutical Chemistry, Faculty of Pharmacy, Horus University-Egypt, New Damietta 34518, Egypt; shawas@horus.edu.eg; 3 Department of Biochemistry, Faculty of Pharmacy, Delta University for Science and Technology, Gamasa 11152, Egypt; dr.mohamed.salama@hotmail.com; 4 Department of Histology, Faculty of medicine (Boys), Al-Azhar University, Cairo 11651, Egypt; histology3.jed@bmc.edu.sa; 5 Department of Histology, General Medicine Practice Program, Batterjee Medical College, Jeddah 21442, Saudi Arabia; 6 Department of Medical Physiology, Faculty of Medicine, Cairo University, Cairo 12613, Egypt; hader.sakr@bmc.edu.sa; 7 Department of Physiology, General Medicine Practice Program, Department of Medical Physiology, Batterjee Medical College, Jeddah 21442, Saudi Arabia; 8 Department of Pharmacy Practice, College of Pharmacy, Princess Nourah bint Abdulrahman University, Riyadh 11671, Saudi Arabia; 9 Department of Pharmacology and Toxicology, Faculty of Pharmacy (Girls), Al-Azhar University, Cairo 11651, Egypt; aalzokaky@horus.edu.eg; 10 Department of Pharmacology and Biochemistry (Pharmacology), Faculty of Pharmacy, Horus University-Egypt, New Damietta 34518, Egypt

**Keywords:** ulcerative colitis, norcantharidin, mesalazine, epigenetic, AMPK, 5-aminosalicylic acid

## Abstract

Ulcerative colitis (UC) is a chronic inflammatory disorder associated with cytokine imbalance, epigenetic alterations, and metabolic–redox dysfunction. Despite the widespread use of mesalazine (5-ASA), therapeutic limitations remain. Norcantharidin (NCTD), a synthetic cantharidin analogue, may provide multi-target protection against UC. Experimental colitis was induced in male Sprague-Dawley rats by intrarectal administration of 4% acetic acid (AA). Rats received oral NCTD (10 mg/kg), 5-ASA (100 mg/kg), or their combination for 8 days. Disease severity was assessed by disease activity index, body weight, colon length, colon weight/length ratio, and histopathology. Colonic biomarkers were evaluated using ELISA, qRT-PCR, Western blotting, and immunohistochemistry. Fe^2+^ and malondialdehyde (MDA) were measured as indicators of iron accumulation and lipid peroxidation. Molecular docking suggested that NCTD may adopt plausible binding poses within the binding pockets of AMPK, SIRT1, and DNMT1, providing structural support for potential protein–ligand interactions. NCTD significantly ameliorated AA-induced colitis, improving clinical and histological outcomes. These effects were associated with reduced IL-6, TNF-α, DNMT1, Fe^2+^, and MDA levels, restoration of SOCS3, activation of p-AMPK/SIRT1/FOXO3a signaling, and enhancement of Nrf2/HO-1 defenses. Combined NCTD/5-ASA treatment produced greater clinical and histological protection, with differential effects on molecular markers. Docking studies suggested favorable interactions of NCTD with AMPK, SIRT1, and DNMT1. NCTD treatment was associated with protection against experimental colitis, linked to modulation of inflammatory, epigenetic, metabolic, and antioxidant pathways.

## 1. Introduction

Ulcerative colitis (UC) is a type of inflammatory bowel disease (IBD) characterized by chronic inflammation and destruction of the colonic and rectal mucosa. Common symptoms include abdominal pain, cramps, rectal pain, bloody diarrhea, weight loss, fever, the presence of pus in the stool, nausea, vomiting, mouth ulcers, arthritis, and delayed growth in children [1]. The exact etiology and pathogenesis underlying UC remain unclear. However, genetic factors, nutritional factors, various infections, oxidative stress, disturbed redox balance, gut microbiota imbalance and immunological responses among other pathological variables are all linked to UC [2,3]. A major contributor to UC pathogenesis is the imbalance between proinflammatory and anti-inflammatory mediators [4]. Experimentally induced models of UC aim at gaining a deeper recognition of the mechanisms underlying the disease and scouting for new treatments. Intrarectal acetic acid (AA) administration is a robust and reproducible experimental model for mimicking UC in experimental animals where early intestinal mucosal injury leads to acute colitis [5,6].

Interleukin-6 (IL-6), a member of the interleukin family, plays a crucial role in the inflammatory process [7,8]. During inflammation triggered by infection or injury, IL-6 production rapidly increases and induces the acute phase protein C-reactive protein (CRP). The complex formed by IL-6 and interleukin-6 receptor (IL-6R) binds to the signaling membrane protein glycoprotein 130 (gp130), initiating intracellular signal transduction. Studies indicate a deep involvement of IL-6 in the pathogenesis of IBD, with disease severity correlating with IL-6 concentration [9]. Patients with IBD exhibit significantly elevated IL-6 levels in the colon compared to control [10]. Recent randomized clinical data suggest that selective inhibition of IL-6 trans-signaling can improve clinical response and mucosal outcomes in active UC [11].

Several key cellular mechanisms become dysregulated in UC, exacerbating the disease. For instance, sirtuin-1 (SIRT1) normally helps to regulate inflammation and oxidative stress in the gut. However, in UC, the protective functions of SIRT1 are often overwhelmed, leading to unchecked inflammatory responses and tissue damage [12,13]. Another critical player is adenosine monophosphate-activated protein kinase (AMPK), an energy sensor that, under normal conditions, helps maintain cellular energy balance and promotes autophagy. AMPK activity has been reported to be reduced in models of intestinal and other mucosal inflammatory conditions [14,15], which may contribute to the accumulation of cellular debris and further aggravate inflammation.

Norcantharidin (NCTD), a synthetic analogue of cantharidin isolated from Mylabris [16], has attracted considerable attention due to its potent anti-inflammatory and pro-apoptotic activities [17]. Previous studies have demonstrated that NCTD exerts beneficial effects in various experimental disease models by attenuating inflammatory responses and modulating key signaling pathways involved in immune regulation and tissue injury [18,19,20]. Despite these promising pharmacological properties, its therapeutic potential in UC remains largely unexplored.

In particular, whether NCTD can regulate the interconnected IL-6/DNMT1/SOCS3 epigenetic–inflammatory axis and the AMPK/SIRT1/FOXO3a-Nrf2 metabolic–redox signaling pathway, both critically implicated in UC pathogenesis [21,22], has not yet been investigated in experimental colitis. Therefore, the present study was designed to evaluate the protective effects of NCTD in AA-induced colitis and to elucidate the underlying molecular mechanisms, either alone or in combination with 5-ASA. To our knowledge, this is the first study to examine NCTD in experimental UC, characterizing the combined IL-6/DNMT1/SOCS3 and AMPK/SIRT1/FOXO3a-Nrf2/HO-1 signaling pathways. We differentiate this from the prior DSS-model cantharidin analogue study [23], which focused on NF-κB-mediated cytokine suppression, by extending mechanistic characterization to epigenetic, metabolic, and antioxidant signaling networks.

## 2. Results

### 2.1. Norcantharidin Attenuates Experimental Colitis Severity

**Figure 1:**
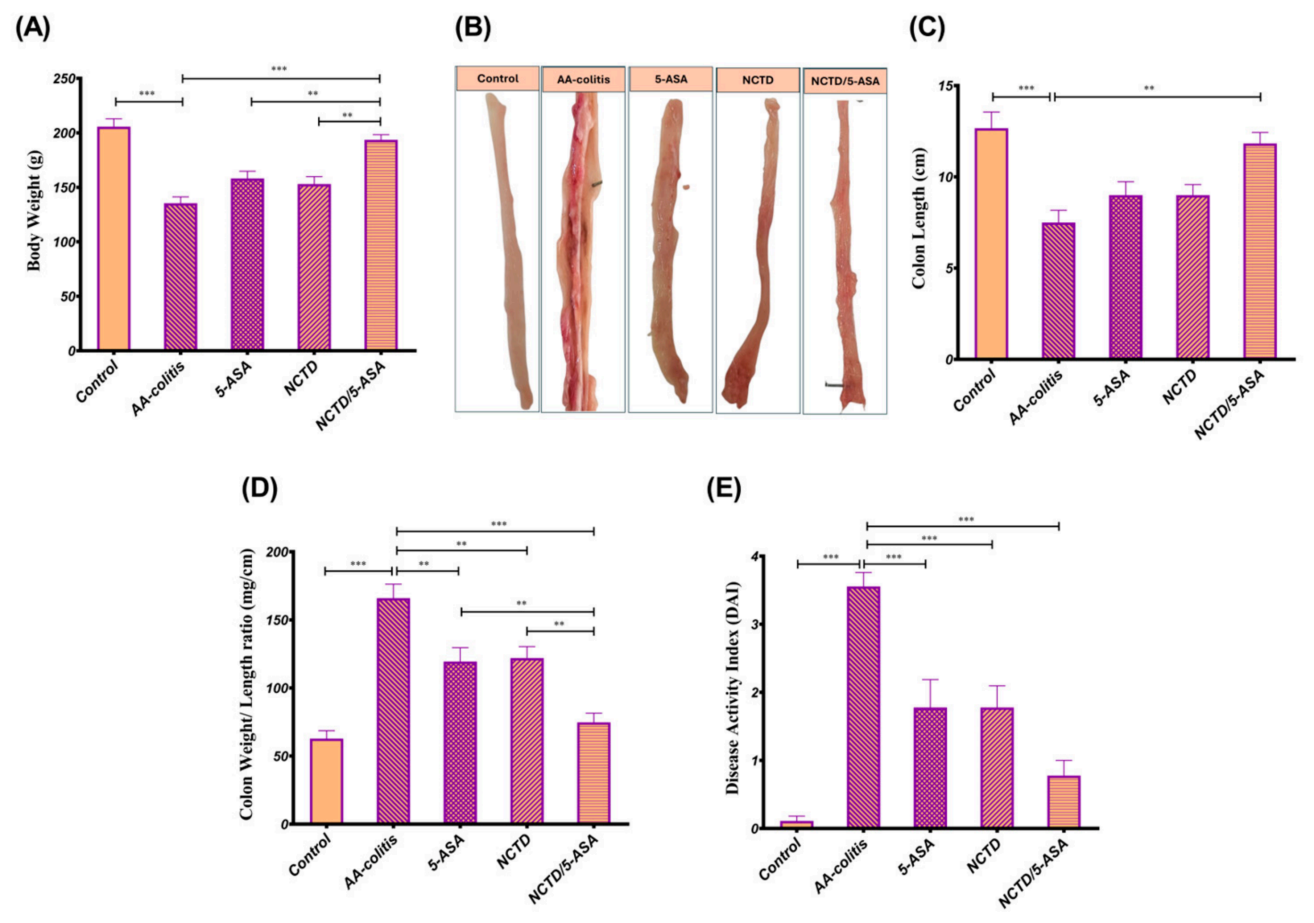
Norcantharidin ameliorates clinical and gross morphometric manifestations of acetic acid-induced colitis in rats. Effects of mesalazine (5-ASA), norcantharidin (NCTD), and their combination on (**A**) body weight, (**B**) representative gross appearance of the colon, (**C**) colon length, (**D**) colon weight/length (W/L) ratio, and (**E**) disease activity index (DAI) in rats with acetic acid (AA)-induced colitis. Data are presented as mean ± SE (*n* = 6). ** *p* ˂ 0.01 and *** *p* ˂ 0.001.

AA administration induced severe colitis, as evidenced by bloody diarrhea, marked body weight loss, hemorrhagic and ulcerated colonic mucosa, colon shortening, an increased colon W/L ratio, and a marked elevation in DAI. Relative to the control group, the AA-colitis group exhibited 34.11% reduction in body weight (*p* < 0.001), 40.8% decrease in colon length (*p* ˂ 0.001), 164.3% increase in colon W/L ratio (*p* ˂ 0.001), and 32-fold increase in DAI (*p* ˂ 0.001). Treatment with NCTD or 5-ASA ameliorated these changes, whereas the NCTD/5-ASA combination conferred the most pronounced protection. Specifically, combined treatment increased body weight by 42.9% compared with the AA-colitis group (*p* ˂ 0.001) and by 22.4% and 26.6% compared with the 5-ASA and NCTD groups (*p* ˂ 0.01), respectively. It also increased colon length by 57.8% (*p* ˂ 0.01) and reduced the colon W/L ratio by 54.98% (*p* ˂ 0.001) compared with the AA-colitis group, with additional significant reductions relative to both monotherapy groups (37.4% and 38.8%, respectively; *p* ˂ 0.01). Consistent with these findings, DAI was significantly reduced by all treatments, with the combination regimen producing the greatest decline (4.57-fold, *p* ˂ 0.01) as compared with the UC model group. Moreover, the NCTD/5-ASA group exhibited the least visible colonic injury among all colitic groups (Figure 1).

### 2.2. Norcantharidin Ameliorates Histopathological Injury

**Figure 2:**
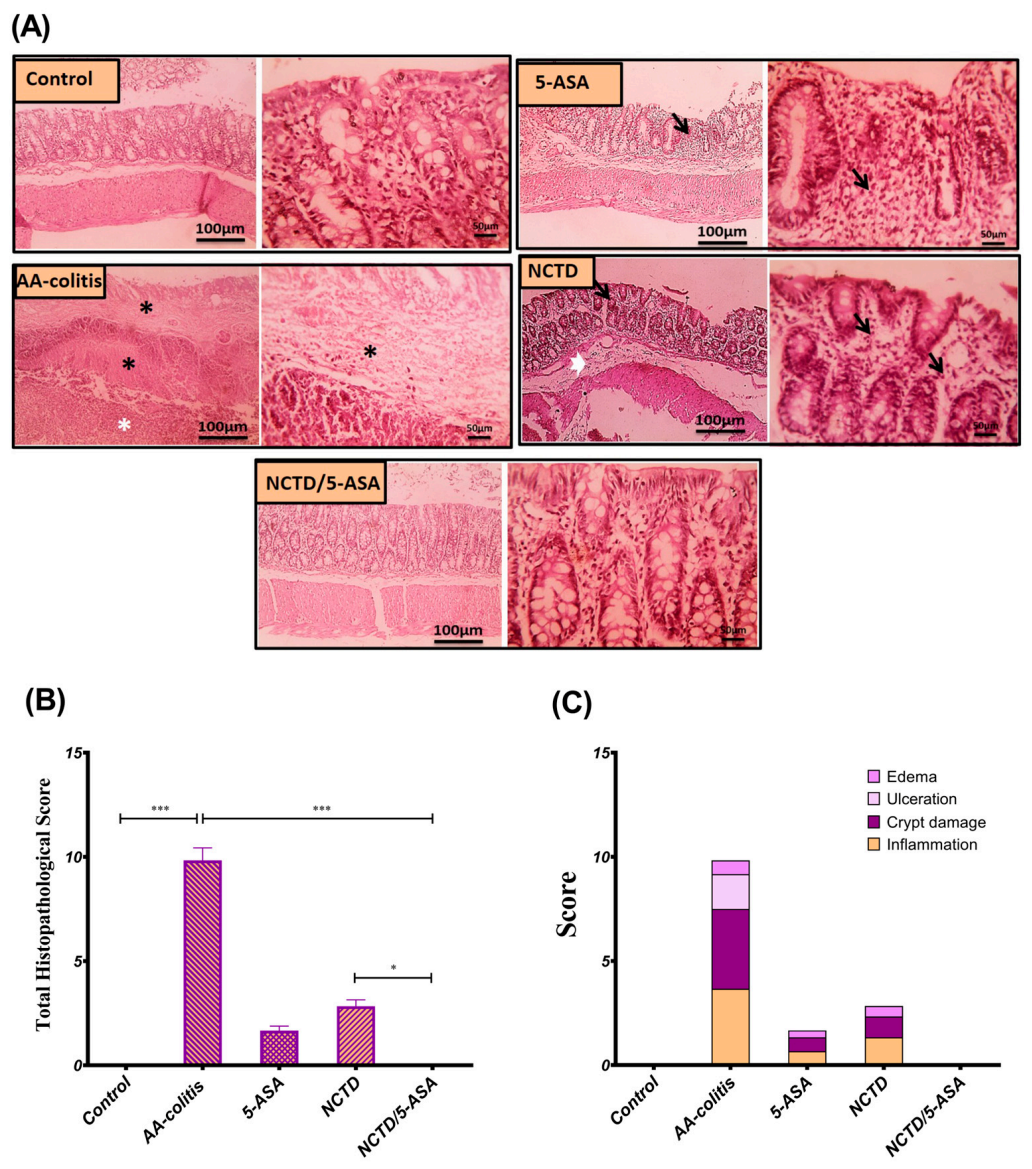
Norcantharidin ameliorates microscopic colonic injury in acetic acid-induced colitis. (**A**) Representative H&E-stained colonic sections from the different experimental groups. The black asterisk indicates severe transmural necrosis, the white asterisk indicates deep severe inflammation, the thin black arrow indicates mild focal mucosal inflammation, and the thick white arrow indicates submucosal edema. Representative images are shown at original magnifications of ×100 and ×400, with scale bars of 100 µm and 50 µm, respectively. (**B**) Total histopathological score. (**C**) Scores for the evaluated histopathological parameters, including inflammatory cell infiltration, crypt damage, ulceration, and edema. Data are presented as mean ± SE (*n* = 6). * *p* ˂ 0.05 and *** *p* ˂ 0.001.

Histological examination of control colon sections revealed preserved mucosal structure with normal crypt organization and intact submucosal and muscular layers. In contrast, AA-induced colitis produced severe transmural necrosis, extensive inflammatory infiltration, and marked crypt loss. Treatment with 5-ASA partially improved these histological abnormalities, with residual focal mucosal inflammation still evident, whereas NCTD treatment reduced tissue injury but remained associated with mild diffuse mucosal and submucosal edema. Notably, NCTD/5-ASA combination resulted in the most evident restoration of colonic structure, with marked preservation of mucosal and submucosal integrity. This qualitative improvement was supported by a significant reduction in total histopathological score relative to the AA-colitis group, together with attenuation of the individual injury components, including inflammatory infiltration, crypt damage, ulceration, and edema (Figure 2).

### 2.3. Norcantharidin Suppresses Colonic Inflammatory Cytokine Responses

**Figure 3:**
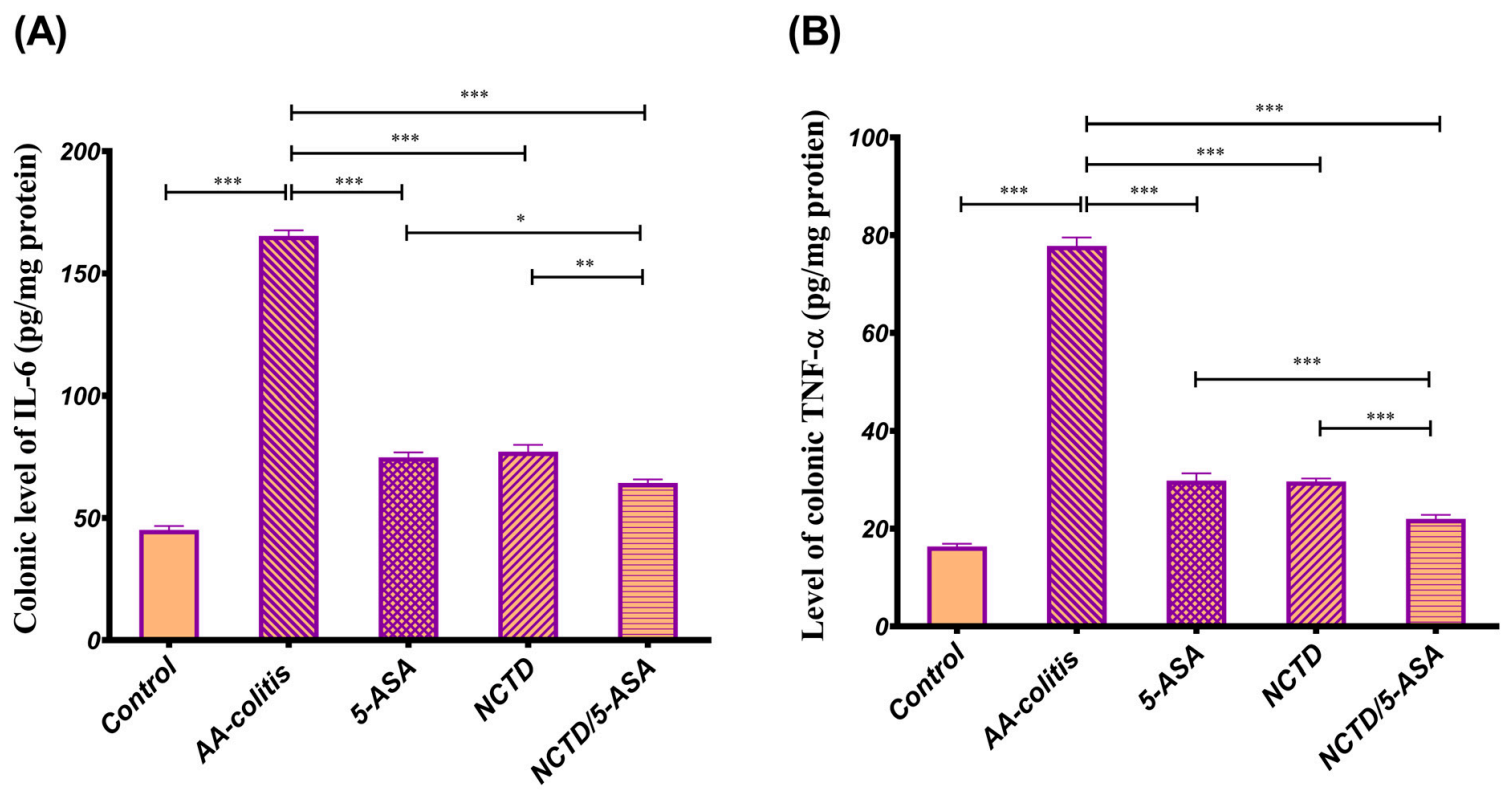
Norcantharidin attenuates colonic inflammation in acetic acid-induced colitis. Effects of mesalazine (5-ASA), norcantharidin (NCTD), and their combination on colonic levels of (**A**) interleukin-6 (IL-6) and (**B**) tumor necrosis factor-α (TNF-α) in rats with acetic acid-induced colitis. Cytokine levels are expressed as pg/mg protein. Data are presented as mean ± SE (*n* = 6). * *p* < 0.05, ** *p* < 0.01, and *** *p* < 0.001.

AA-induced colitis was associated with a marked inflammatory response, as reflected by significant increases in colonic IL-6 and TNF-α levels by 3.7-fold and 4.8-fold (*p* ˂ 0.001), respectively, relative to controls. Both NCTD and 5-ASA significantly reduced these cytokines, whereas the combination treatment exerted the greatest anti-inflammatory effect. Compared with the AA-colitis group, NCTD/5-ASA reduced IL-6 and TNF-α by 2.6-fold and 3.5-fold (*p* ˂ 0.001), respectively. Moreover, combined treatment produced a further, modest improvement over either monotherapy, lowering IL-6 by 1.16-fold (*p* ˂ 0.05) and 1.2-fold (*p* ˂ 0.01) relative to the 5-ASA and NCTD groups, respectively, and reducing TNF-α by 1.35-fold (*p* ˂ 0.001) relative to the monotherapy-treated groups. These findings indicate that NCTD, particularly when combined with 5-ASA, effectively suppresses the cytokine response associated with AA-induced colonic inflammation (Figure 3).

### 2.4. Molecular Docking Suggests Potential Interactions of Norcantharidin with AMPK, SIRT1, and DNMT1

Molecular docking studies were performed to investigate the potential interactions of NCTD with AMPK (4CFE), SIRT1 (5BTR), and DNMT1 (7SFC). The docking analyses compared the binding mode of NCTD with the respective co-crystallized ligands to better understand the potential interactions contributing to its activity. The docking protocol was validated by redocking the co-crystallized ligand into the corresponding binding site, yielding RMSD values of 1.749, 0.534, and 1.605 Å for AMPK, SIRT1, and DNMT1, respectively, confirming the reliability of the docking procedure.

**Figure 4:**
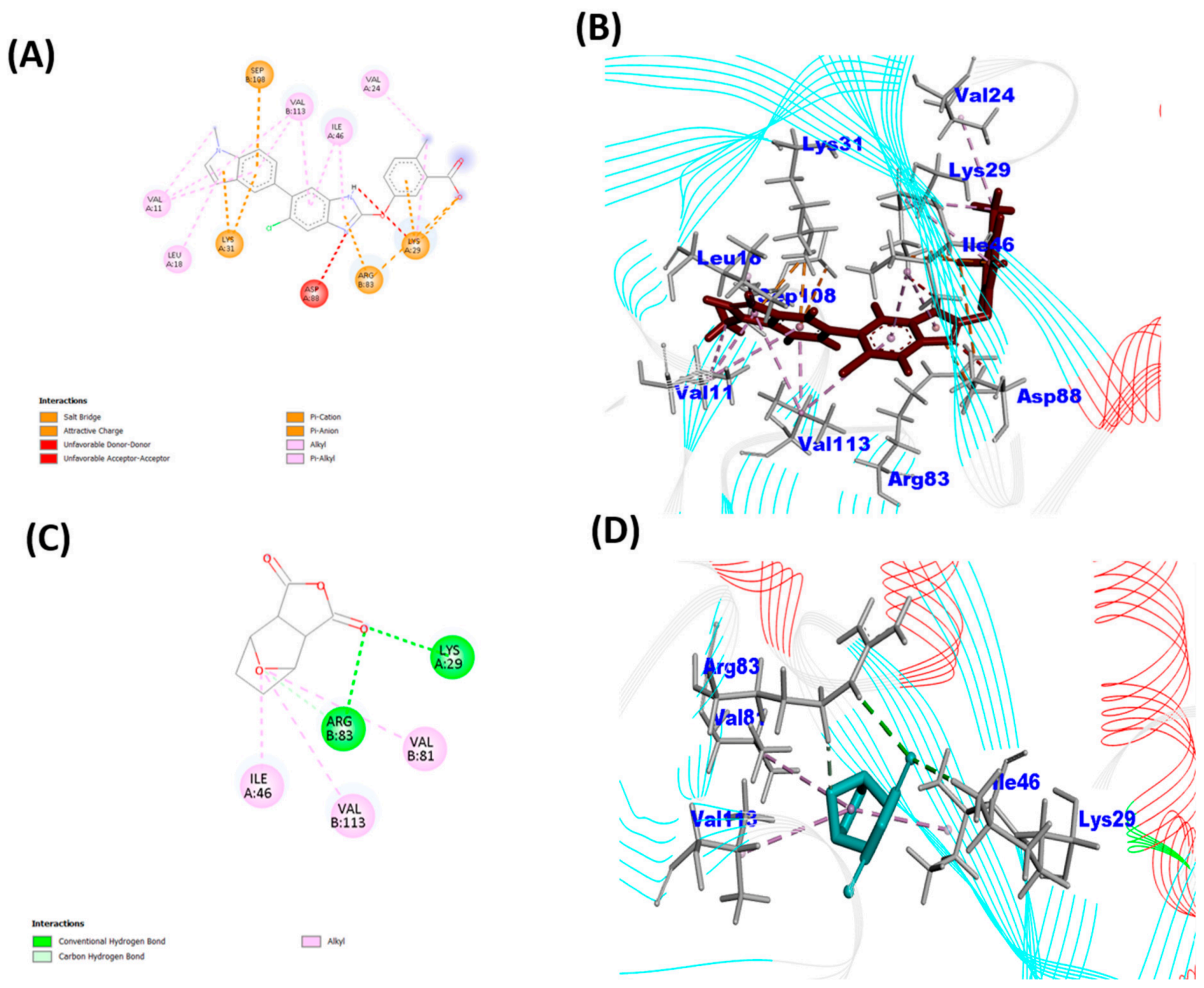
Docking analysis of norcantharidin within the binding pocket of the 4CFE crystal structure. (**A**) Two-dimensional interaction map of the co-crystallized ligand. (**B**) Three-dimensional binding pose of the co-crystallized ligand (brown) within the binding site. (**C**) Two-dimensional interaction map of norcantharidin. (**D**) Three-dimensional binding pose of norcantharidin (light blue) within the binding pocket.

In the AMPK (PDB: 4CFE) structure, the co-crystallized ligand exhibited several hydrophobic interactions, including alkyl contacts with VAL11, LEU18, VAL113, ILE46, and VAL24. In addition, strong electrostatic interactions were observed through salt bridges with LYS31, LYS29, and ARG83, while a donor–donor interaction was detected with ASP88. Docking of NCTD showed comparable interactions within the binding pocket, forming alkyl interactions with ILE46, VAL113, and VAL81 with a score of −5.9 kcal/mol. The redocked co-crystallized ligand exhibited a docking score of −10.6 kcal/mol, providing a reference for comparison with the predicted docking score of NCTD (−5.9 kcal/mol). NCTD retained interactions with key active-site residues, supporting a plausible binding mode within the binding site. Furthermore, hydrogen bonds were observed with LYS29 and ARG83, suggesting that NCTD may occupy a similar region of the binding pocket and establish predicted interactions with key residues (Figure 4).

**Figure 5:**
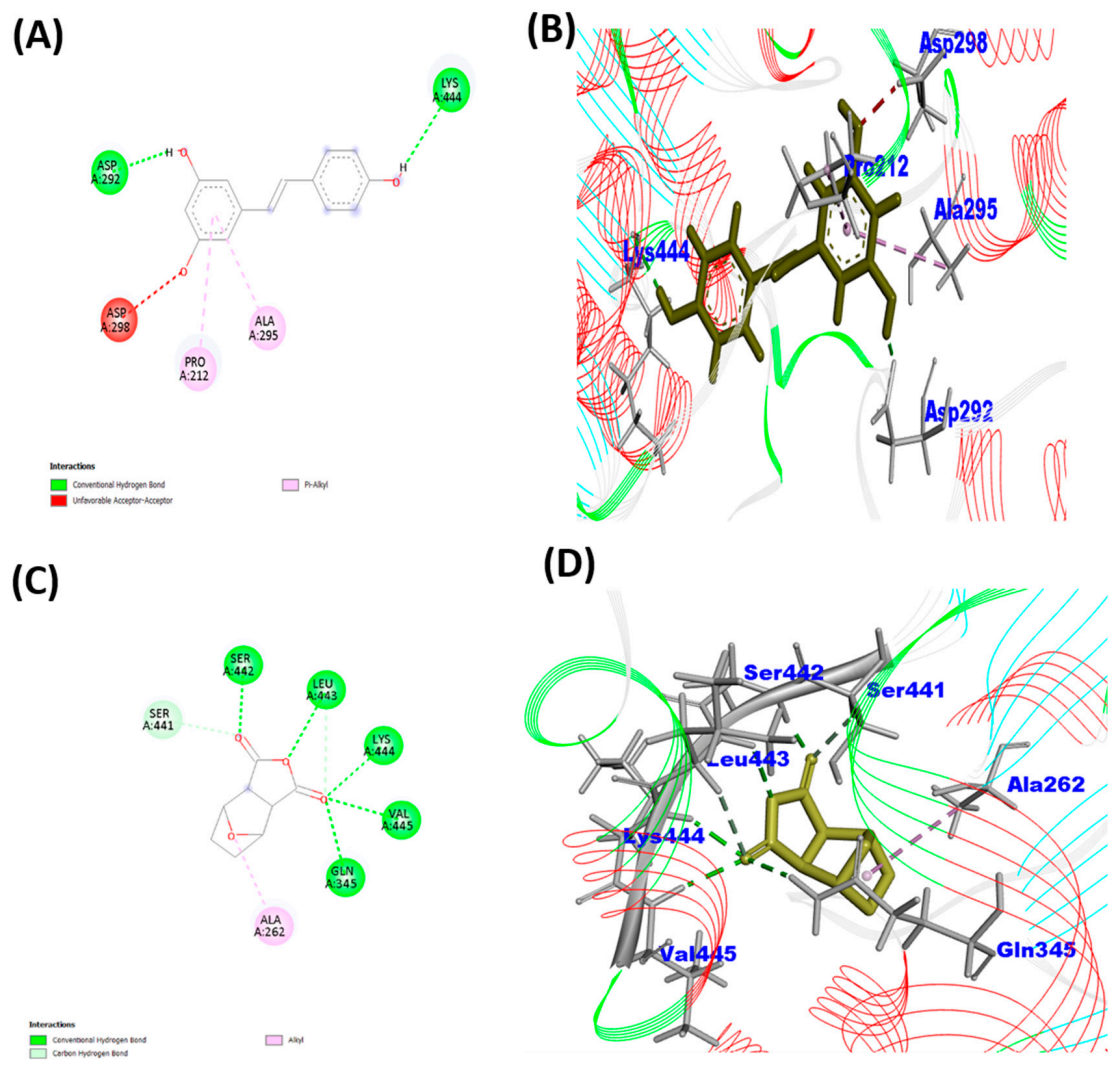
Docking analysis of norcantharidin within the binding pocket of the 5BTR crystal structure. (**A**) Two-dimensional interaction map of the co-crystallized ligand. (**B**) Three-dimensional binding pose of the co-crystallized ligand (gold) within the binding site. (**C**) Two-dimensional interaction map of norcantharidin. (**D**) Three-dimensional binding pose of norcantharidin (yellow) within the binding pocket of the 5BTR structure.

In the SIRT1 (PDB: 5BTR) crystal structure, the co-crystallized ligand established hydrogen bonds with ASP292 and LYS444, along with hydrophobic alkyl interactions with PRO212 and ALA295. An additional acceptor–acceptor interaction was noted with ASP298. Docking results for NCTD revealed multiple hydrogen bonding interactions with SER441, LEU443, LYS444, VAL445, and GLN345, as well as a hydrophobic alkyl interaction with ALA262 with a predicted docking score of −6.5 kcal/mol. The redocked co-crystallized ligand exhibited a docking score of −6.7 kcal/mol. These predicted interactions provide structural support for a plausible binding mode of NCTD within the SIRT1 binding pocket (Figure 5).

**Figure 6:**
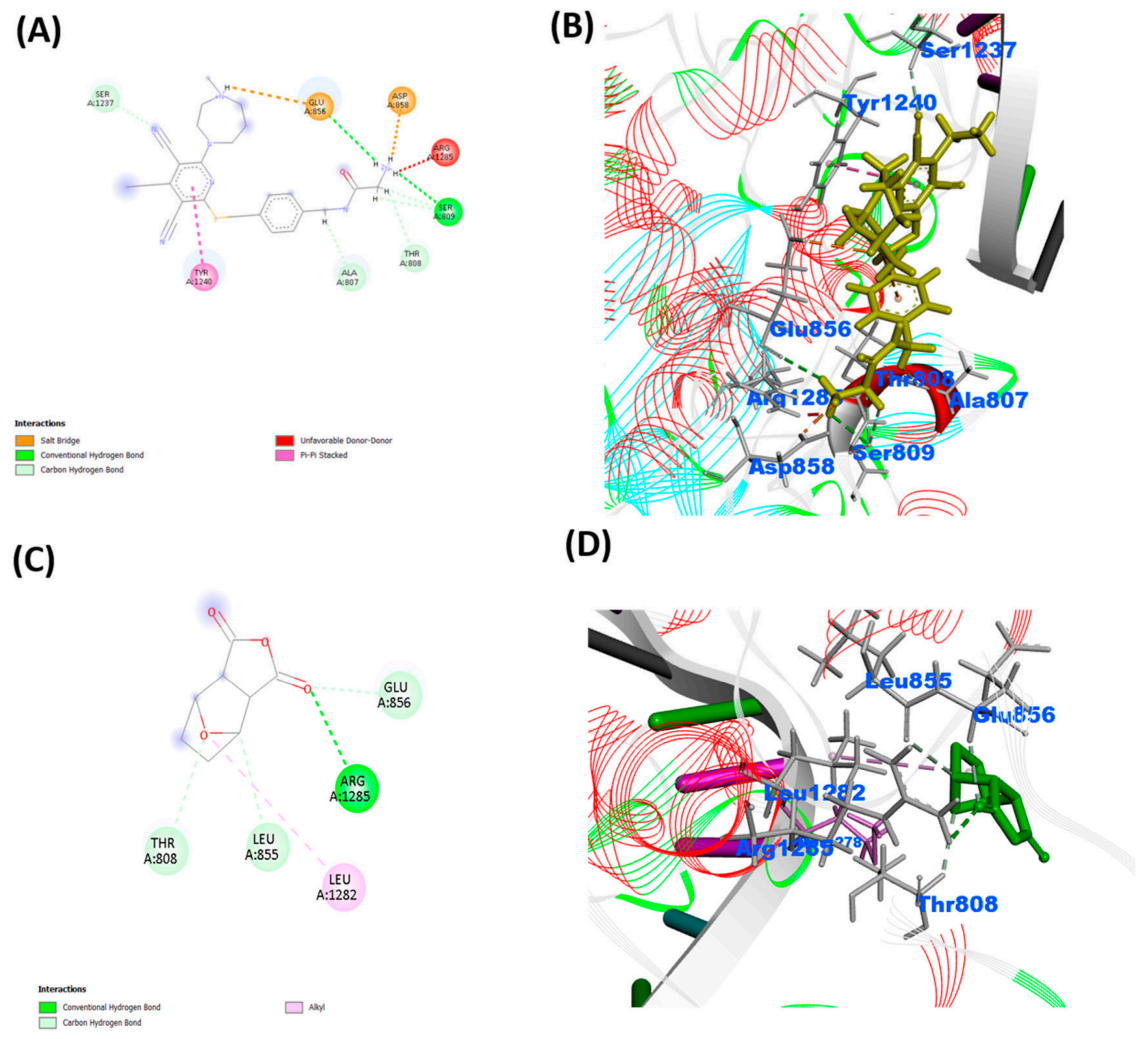
Docking analysis of norcantharidin within the binding pocket of the 7SFC crystal structure. (**A**) Two-dimensional interaction map of the co-crystallized ligand. (**B**) Three-dimensional binding pose of the co-crystallized ligand (yellow) within the enzyme binding pocket. (**C**) Two-dimensional interaction map of norcantharidin, highlighting hydrogen-bonding interactions. (**D**) Three-dimensional binding pose of norcantharidin (green) within the binding site of the 7SFC structure.

For the DNMT1 (PDB:7SFC) structure, the co-crystallized ligand demonstrated several important interactions, including π–π stacking with TYR1240, hydrogen bonds with SER1237, ALA807, THR808, and SER809, salt bridges with GLU856 and ASP858, and a donor–donor interaction with ARG1285. Docking of NCTD showed hydrogen bond formation with THR808, LEU855, GLU856, and ARG1285, along with an alkyl interaction with LEU1282 with a predicted docking score of −4.4 kcal/mol. The redocked co-crystallized ligand exhibited a docking score of −7.0 kcal/mol. These predicted interactions support a plausible binding orientation of NCTD within the DNMT1 binding pocket (Figure 6).

Overall, the docking results suggest that NCTD may adopt plausible binding poses within the binding pockets of AMPK, SIRT1, and DNMT1 and establish hydrogen-bonding and hydrophobic interactions with key amino acid residues. These computational findings provide structural support for potential protein–ligand interactions and complement the experimental observations; however, they should be interpreted as mechanistic hypotheses rather than direct evidence of agonistic or inhibitory activity.

### 2.5. Norcantharidin Modulates DNMT1/SOCS3 and AMPK/SIRT1/FOXO3a Signaling

**Figure 7:**
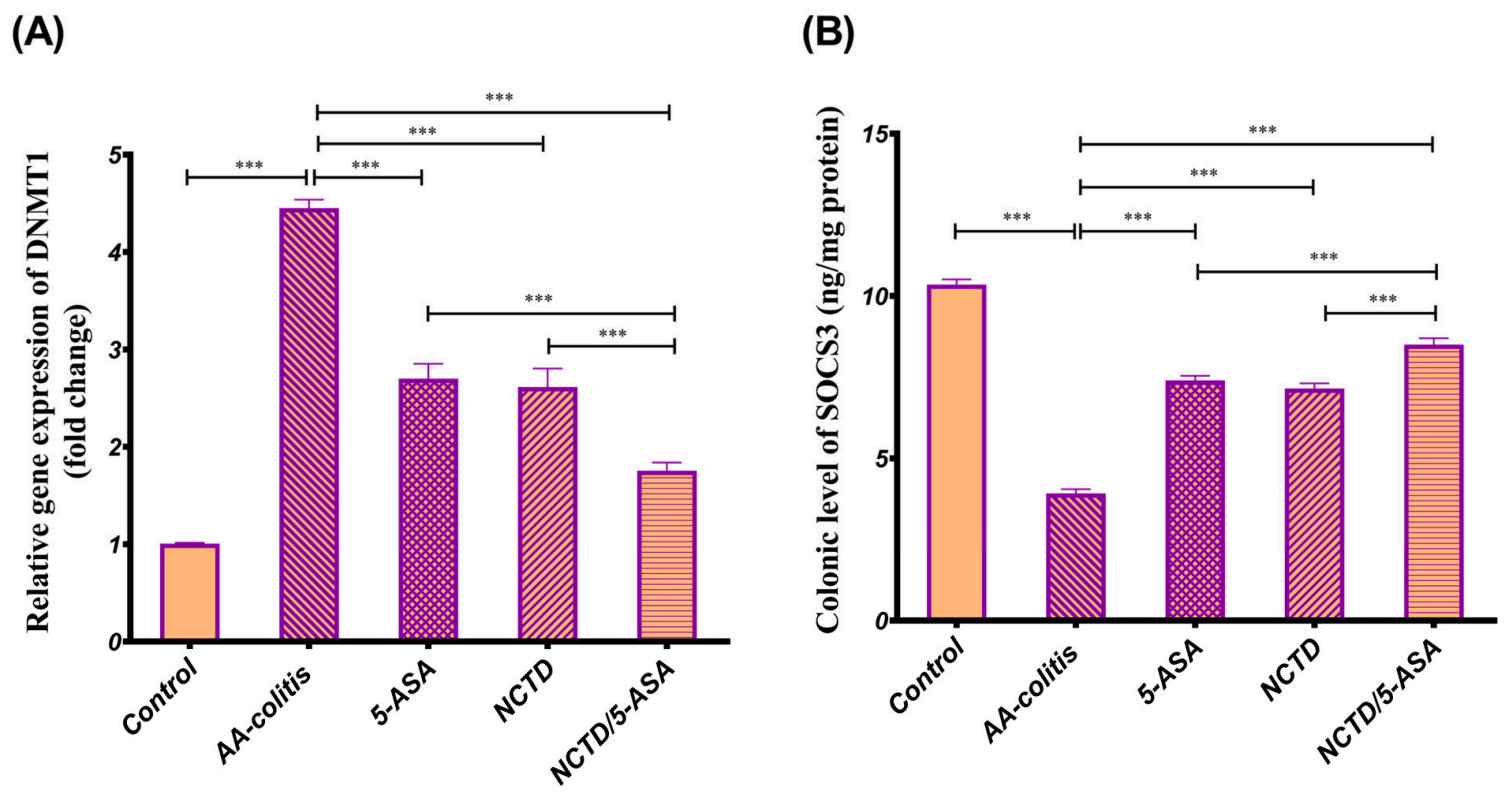
Norcantharidin modulates the DNMT1/SOCS3 pathway in colonic tissue of rats with acetic acid-induced colitis. Effects of mesalazine (5-ASA), norcantharidin (NCTD), and their combination on colonic (**A**) DNMT1 mRNA expression and (**B**) SOCS3 protein levels in rats with acetic acid-induced colitis. SOCS3 levels are expressed as ng/mg protein. Data are presented as mean ± SE (*n* = 6). *** *p* ˂ 0.001.

To define the molecular basis of NCTD-mediated protection, the DNMT1/SOCS3 signaling was evaluated in colonic tissue. AA-induced colitis markedly upregulated DNMT1 expression by 4.42-fold (*p* ˂ 0.001) while reducing SOCS3 levels by 2.6-fold (*p* ˂ 0.001) relative to the control group. Both NCTD and 5-ASA partially corrected this dysregulation, whereas the NCTD/5-ASA combination exerted the most pronounced effect, reducing DNMT1 by 2.54-fold (*p* ˂ 0.001) and increasing SOCS3 by 2.17-fold (*p* ˂ 0.001) compared with the AA-colitis group. Notably, the combination regimen showed a further, modest improvement over both monotherapies, as evidenced by a further reduction in DNMT1 by 1.54-fold and 1.49-fold (*p* ˂ 0.001) relative to the 5-ASA and NCTD groups, respectively, together with a further increase in SOCS3 by 1.15-fold and 1.19-fold (*p* ˂ 0.001), respectively (Figure 7).

**Figure 8:**
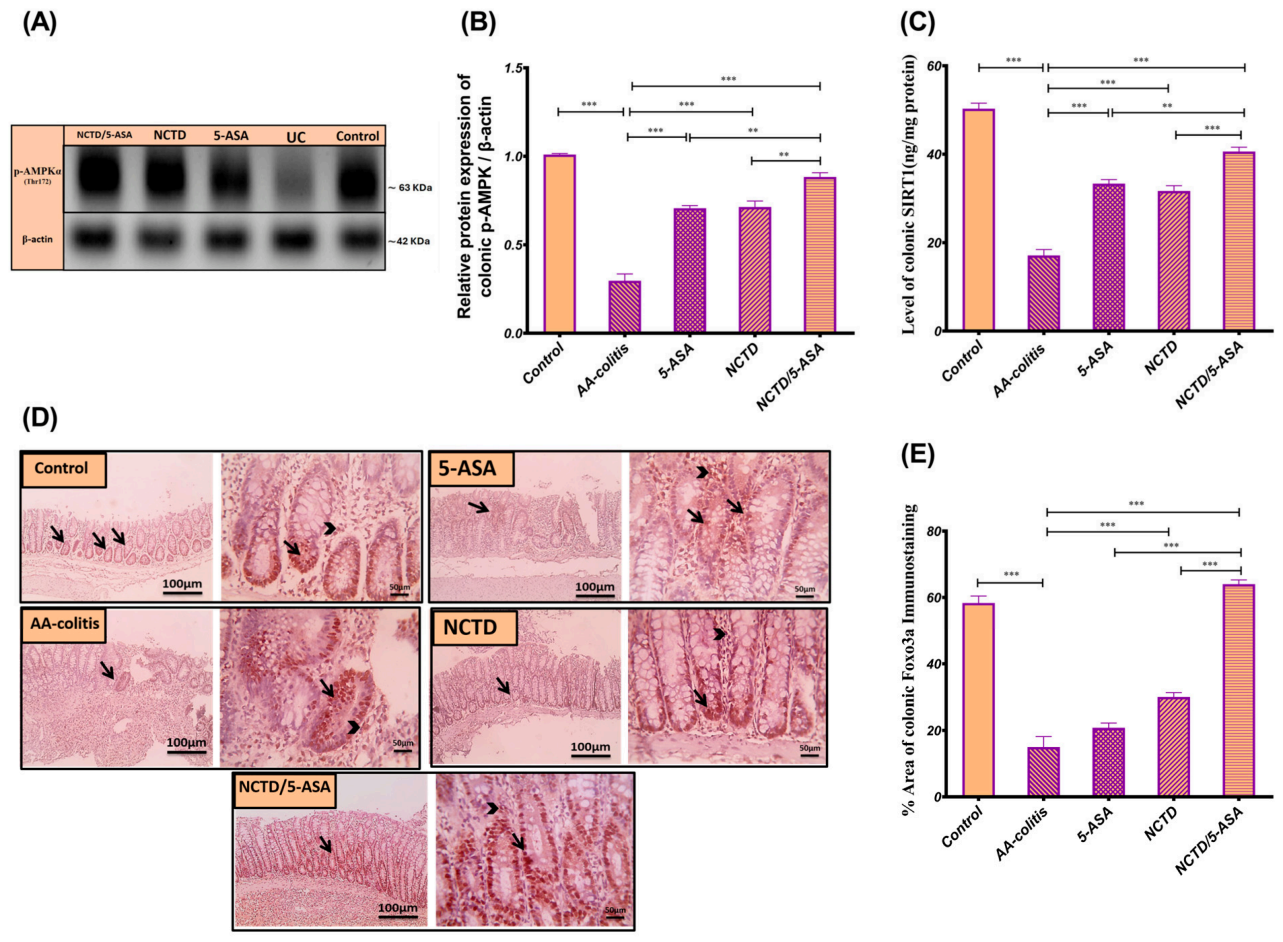
Norcantharidin modulated the AMPK/SIRT1/FOXO3a pathway in colonic tissue of rats with acetic acid-induced colitis. (**A**) Representative Western blot bands of colonic p-AMPK expression in the different groups. (see Supplementary Materials). (**B**) Relative colonic p-AMPK protein expression determined by densitometric analysis. (**C**) Colonic levels of SIRT1 (ng/mg protein). (**D**) Representative FOXO3a-immunostained colon sections. (**E**) Quantification of FOXO3a expression as positive area (%). Data are presented as mean ± SE (*n* = 6). Arrows indicate positive brown nuclear staining in crypt epithelial cells, whereas arrowheads indicate positively stained interstitial cells within the lamina propria. Sections were counterstained with Mayer’s hematoxylin. Representative images are shown at original magnifications of ×100 and ×400, with corresponding scale bars of 100 µm and 50 µm, respectively. ** *p* < 0.01, *** *p* < 0.001.

This mechanistic recovery extended to the AMPK/SIRT1/FOXO3a pathway. AA administration markedly suppressed colonic p-AMPK, SIRT1, and FOXO3a levels by 3.4-fold, 2.9-fold, and 3.89-fold (*p* ˂ 0.001), respectively, relative to the control group. NCTD treatment was associated with restoration of p-AMPK, SIRT1, and FOXO3a levels by 2.4-fold, 1.85-fold, and 2-fold (*p* ˂ 0.001), respectively, whereas 5-ASA significantly increased p-AMPK and SIRT1 levels by 2.38-fold and 1.95-fold (*p* ˂ 0.001), respectively, compared with the AA-colitis group. Notably, the NCTD/5-ASA combination produced the most pronounced effect, increasing p-AMPK, SIRT1, and FOXO3a levels by 2.98-fold, 2.37-fold, and 4.3-fold (*p* ˂ 0.001), respectively, compared with the AA-colitis group. The combination regimen showed further improvement than both monotherapies, as evidenced by further increases in p-AMPK by 1.25-fold and 1.24-fold (*p* ˂ 0.01), SIRT1 by 1.22-fold (*p* ˂ 0.01) and 1.28-fold (*p* ˂ 0.001), and FOXO3a by 3.1-fold and 2.13-fold (*p* ˂ 0.001), relative to the 5-ASA and NCTD groups, respectively. Immunohistochemical analysis further confirmed enhanced FOXO3a expression in the combined-treatment group, consistent with modulation of this cytoprotective signaling pathway (Figure 8).

### 2.6. Norcantharidin Activates Nrf2/HO-1 Signaling and Attenuates Iron Accumulation and Lipid Peroxidation in Colonic Tissue

**Figure 9:**
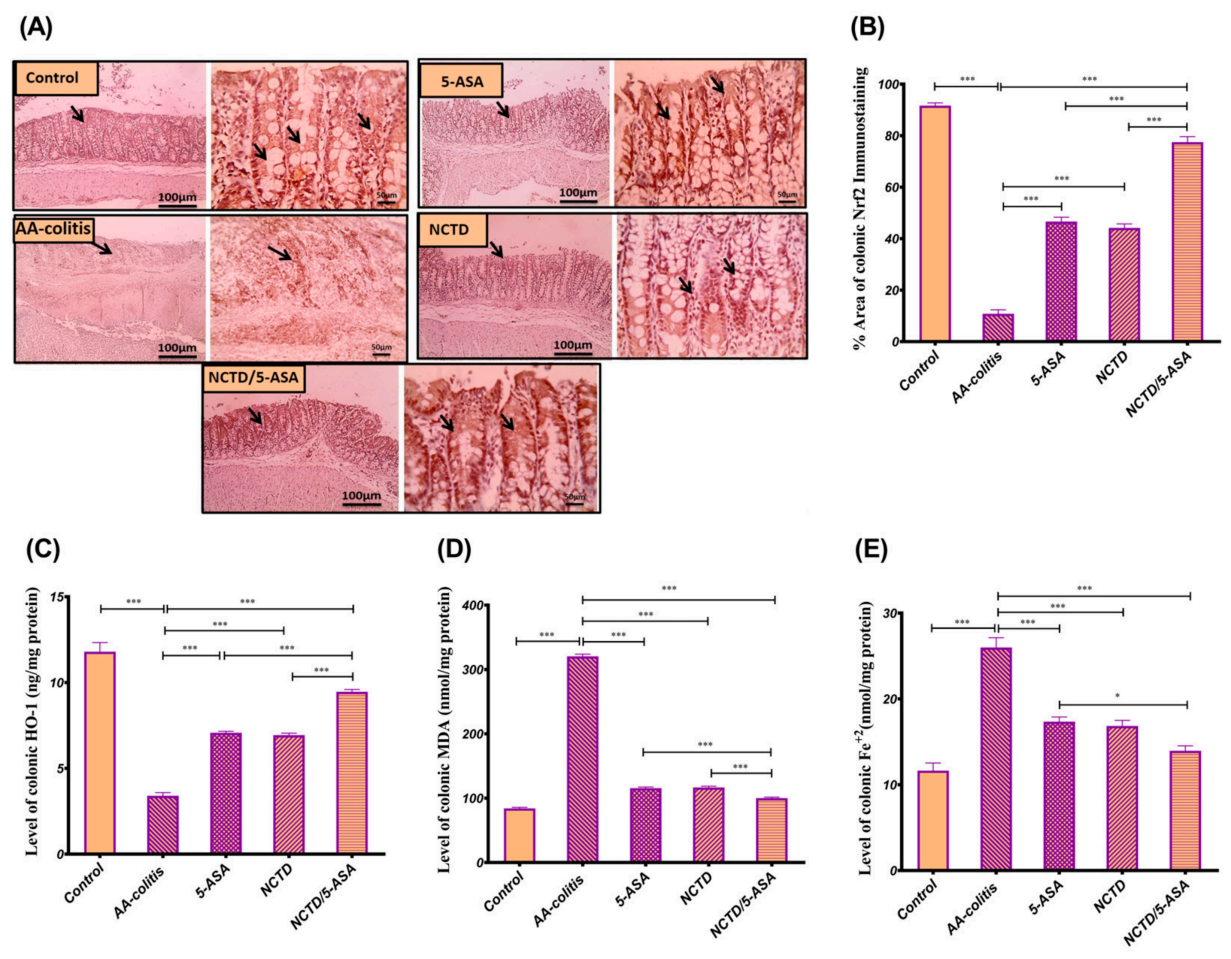
Norcantharidin activates Nrf2/HO-1 signaling and attenuates iron accumulation and lipid peroxidation in colonic tissue. (**A**) Representative Nrf2-immunostained colon sections. (**B**) Quantification of Nrf2 expression as positive area (%). Arrows indicate positive brown nuclear staining in crypt epithelial cells. Sections were counterstained with Mayer’s hematoxylin. Representative images are shown at original magnifications of ×100 and ×400, with corresponding scale bars of 100 µm and 50 µm, respectively. (**C**) Colonic levels of HO-1 (ng/mg protein). (**D**) Colonic levels of MDA (nmol/mg protein). (**E**) Colonic levels of Fe^2+^ (nmol/mg protein). Data are presented as mean ± SE (*n* = 6). * *p* < 0.05, *** *p* < 0.001.

Given that oxidative stress, iron accumulation, and lipid peroxidation may contribute to mucosal damage in AA-induced colitis, the Nrf2/HO-1 axis together with Fe^2+^ and MDA levels was evaluated. AA administration markedly suppressed colonic Nrf2 and HO-1 levels by 8.5-fold and 3.5-fold (*p* ˂ 0.001), respectively, relative to the control group. Both NCTD and 5-ASA significantly restored these antioxidant defenses, whereas the NCTD/5-ASA combination produced the most pronounced effect, increasing Nrf2 by 7.2-fold and HO-1 by 2.8-fold (*p* ˂ 0.001), respectively, compared with the AA-colitis group. The combination regimen showed a further, modest improvement over both monotherapies, as evidenced by further increases in Nrf2 by 1.66-fold and 1.75-fold (*p* ˂ 0.001), and in HO-1 by 1.34-fold and 1.36-fold (*p* ˂ 0.001), relative to the 5-ASA and NCTD groups, respectively (Figure 9A–C).

Consistent with these changes, AA-induced colitis markedly increased colonic Fe^2+^ and MDA levels by 2.2-fold and 3.8-fold (*p* ˂ 0.001), respectively, indicating enhanced iron accumulation and lipid peroxidation. NCTD and 5-ASA each attenuated these alterations in iron accumulation and lipid peroxidation, whereas combined treatment exerted the most substantial effect, reducing Fe^2+^ and MDA by 1.86-fold and 3.2-fold (*p* ˂ 0.001), respectively, compared with the AA-colitis group. The combination also produced additional significant reductions in Fe^2+^ and MDA by 1.24-fold (*p* ˂ 0.05) and 1.16-fold (*p* ˂ 0.001), respectively, relative to the 5-ASA group, and further reduced MDA by 1.17-fold (*p* ˂ 0.001) relative to the NCTD group. Collectively, these findings indicate that combined NCTD/5-ASA treatment provides greater protection against oxidative stress, iron accumulation, and lipid peroxidation in colonic tissue (Figure 9D,E).

## 3. Discussion

UC is a chronic relapsing inflammatory disorder driven by the interplay of immune dysregulation, epithelial barrier disruption, epigenetic alterations, and metabolic imbalance rather than a purely cytokine-mediated disease [24,25,26]. Although 5-ASA remains a cornerstone first-line therapy, a substantial proportion of patients do not achieve sustained remission, highlighting the need for therapeutic strategies that target upstream regulatory networks [27,28,29]. In this context, the present study shows that NCTD, particularly in combination with 5-ASA, ameliorates experimental colitis and is associated with modulation of interconnected inflammatory, epigenetic, metabolic, and redox pathways.

Our findings show that AA-induced colitis resulted in marked clinical and structural injury, as evidenced by body weight loss, diarrhea, colon shortening, and an increased colon weight/length ratio, reflecting substantial inflammation and tissue edema. Treatment with NCTD or 5-ASA improved these parameters, whereas the combination regimen produced the greatest overall benefit and was superior to either monotherapy. This effect extended beyond symptomatic improvement to include marked restoration of mucosal integrity and attenuation of histopathological injury, indicating effective suppression of inflammation together with enhanced tissue recovery. These findings are consistent with previous reports in experimental colitis, in which many interventions improve selected inflammatory parameters but achieve only partial restoration of tissue structure [30,31,32]. Collectively, these findings suggest that modulation of multiple pathogenic pathways was associated with greater therapeutic benefit in the combination-treatment group than single-agent treatment.

A central finding of this study is that NCTD treatment was associated with modulation of the IL-6/DNMT1/SOCS3 signaling axis, an epigenetic–inflammatory pathway implicated in UC pathogenesis. Consistent with prior reports, IL-6 was markedly elevated in AA-induced colitis [33,34,35,36]. While IL-6 is well established as a key driver of UC severity and STAT3 activation [37,38], emerging evidence suggests that sustained IL-6 signaling promotes DNMT1-mediated epigenetic silencing of SOCS3, thereby reinforcing a pathogenic feed-forward loop [39,40,41]. Importantly, DNMT1 is a cytokine-responsive epigenetic regulator whose expression can be rapidly induced by IL-6 during inflammatory responses, preceding more stable DNA methylation changes [42,43,44]. In line with previous findings, we observed DNMT1 upregulation accompanied by near-complete loss of SOCS3 expression in colitic tissue [21,45,46]. Importantly, NCTD was associated with reduced DNMT1 expression and increased SOCS3 levels, findings that are consistent with modulation of regulatory mechanisms involved in cytokine signaling. However, because SOCS3 promoter methylation and DNMT1 enzymatic activity were not directly assessed, our results do not establish a causal epigenetic mechanism. Moreover, given the acute nature of the AA-induced colitis model, these findings should be interpreted as evidence of early regulation of the epigenetic machinery rather than definitive or sustained epigenetic remodeling. Rather, they provide indirect evidence that is compatible with the previously proposed IL-6/DNMT1/SOCS3 regulatory pathway. Previous studies have also demonstrated that NCTD suppresses IL-6 signaling in different experimental models [17,47], supporting the possibility that attenuation of IL-6 signaling may contribute, at least in part, to the observed changes in DNMT1 and SOCS3 expression.

In parallel, NCTD exerted profound effects on metabolic and redox homeostasis, highlighting the increasingly recognized role of immunometabolism in UC pathogenesis. Previous studies have demonstrated that NCTD activates AMPK [48,49], while extensive evidence indicates that AMPK activation alleviates experimental colitis through activation of SIRT1 and its downstream antioxidant signaling pathways [50,51,52,53,54,55,56,57,58]. Consistent with these reports, AA-induced colitis was associated with marked reductions in p-AMPK, SIRT1, FOXO3a, Nrf2, and HO-1, whereas NCTD treatment significantly restored their expression. SIRT1-mediated activation of FOXO3a enhances antioxidant defense, while activation of the Nrf2/HO-1 pathway protects against oxidative stress and suppresses inflammatory signaling [15,22,59,60,61,62]. Accordingly, the coordinated restoration of p-AMPK, SIRT1, FOXO3a, Nrf2, and HO-1 observed in the present study, together with the attenuation of oxidative stress and inflammatory indices, is consistent with the established cytoprotective role of this signaling cascade in experimental colitis. Nevertheless, the present findings demonstrate molecular associations rather than direct pathway dependence. Therefore, although our data support the involvement of the IL-6/DNMT1/SOCS3 and AMPK/SIRT1/FOXO3a/Nrf2 signaling pathways in the protective effects of NCTD during acute experimental colitis, future studies employing chronic colitis models together with pathway-specific inhibitors, genetic manipulation, promoter methylation analyses, and rescue experiments are required to determine whether these early molecular changes translate into sustained epigenetic remodeling and to establish causal relationships.

A notable finding associated with these metabolic and redox changes was the reduction in iron accumulation and lipid peroxidation, processes that have been linked to intestinal epithelial injury and have been implicated in UC pathogenesis [61,63,64,65]. Although recent studies have implicated the potential involvement of ferroptosis in UC, its relationship with upstream metabolic and epigenetic regulators remains incompletely understood [64,65]. In the present study, AA-induced colitis was associated with increased colonic Fe^2+^ and MDA levels, reflecting enhanced iron accumulation and oxidative lipid damage, both of which were markedly attenuated by NCTD treatment. These effects were accompanied by restoration of Nrf2/HO-1 signaling, suggesting improved antioxidant defense and redox homeostasis. While increased Fe^2+^ and MDA levels are biochemical changes frequently associated with ferroptosis, they are not specific indicators of this form of regulated cell death. Therefore, our findings demonstrate that NCTD mitigates oxidative tissue injury and reduces indices of iron accumulation and lipid peroxidation in experimental colitis, but they do not provide definitive evidence of ferroptosis inhibition. Future studies incorporating canonical ferroptosis markers, including GPX4, SLC7A11, and ACSL4, together with pharmacological rescue experiments using ferroptosis-specific inhibitors, are warranted to clarify the contribution of ferroptosis-related mechanisms to the protective effects of NCTD.

The combination produced the most pronounced benefit in clinical and histological outcomes. Both 5-ASA and NCTD produced convergent effects on downstream inflammatory and oxidative parameters. 5-ASA acts on downstream inflammatory mediators, including NF-κB signaling, eicosanoid synthesis, and PPARγ activation, thereby reducing TNF-α and IL-6 production and partially restoring redox balance [66,67,68]. NCTD was additionally associated with modulation of upstream epigenetic and metabolic targets, including reduced DNMT1 expression, increased SOCS3 levels, and enhancement of p-AMPK/SIRT1 signaling markers, together with improved antioxidant defenses. The distinct molecular changes associated with NCTD and 5-ASA treatment may contribute to the broader therapeutic effects observed with the combination regimen; however, the relative contribution of each mechanism and their potential interaction require further investigation [21,50]. Such an approach is consistent with emerging therapeutic strategies in UC, in which mechanistically distinct agents are combined to overcome therapeutic resistance and achieve more durable remission [69,70,71,72]. Accordingly, the NCTD/5-ASA regimen combined suppression of inflammatory mediators with modulation of multiple pathways implicated in UC pathogenesis, resulting in the most pronounced improvements in mucosal healing, cytokine burden, and markers of iron accumulation and lipid peroxidation compared with either monotherapy.

The single prior report of a cantharidin derivative in experimental colitis evaluated a structurally modified analogue, rather than NCTD itself, in a DSS-induced chronic inflammation model, with mechanistic focus restricted to NF-κB-mediated cytokine suppression [23]. In contrast, the present study provides the first evidence that NCTD treatment was associated with amelioration of UC-like injury induced by acetic acid, a model characterized by acute mucosal disruption and oxidative stress dominance rather than the prolonged epithelial barrier injury typical of dextran sodium sulfate (DSS) models [73,74]. Importantly, we extend current knowledge by demonstrating that NCTD acts beyond classical inflammatory pathways, involving coordinated regulation of epigenetic (DNMT1/SOCS3), metabolic (AMPK/SIRT1/FOXO3a), and antioxidant (Nrf2/HO-1) signaling networks, together with reductions in iron accumulation and lipid peroxidation.

Despite these promising findings, several limitations warrant consideration. First, a single fixed dose of NCTD was used; dose-ranging studies are needed to define the therapeutic window. Second, the AA-induced colitis model does not capture the chronic, immune-mediated complexity of human UC [75]. Third, the data are correlative; causal relationships would require genetic or pharmacological inhibition studies [76]. Fourth, SOCS3 promoter methylation and DNMT1 enzymatic activity were not assessed, so DNMT1-mediated epigenetic silencing remains unproven. Finally, systemic safety markers (ALT, AST, urea, creatinine) were not measured; although NCTD is a less toxic cantharidin analogue, this gap limits clinical interpretability. These limitations should be addressed in future studies to establish the long-term safety and therapeutic value of the NCTD/5-ASA combination.

**Figure 10:**
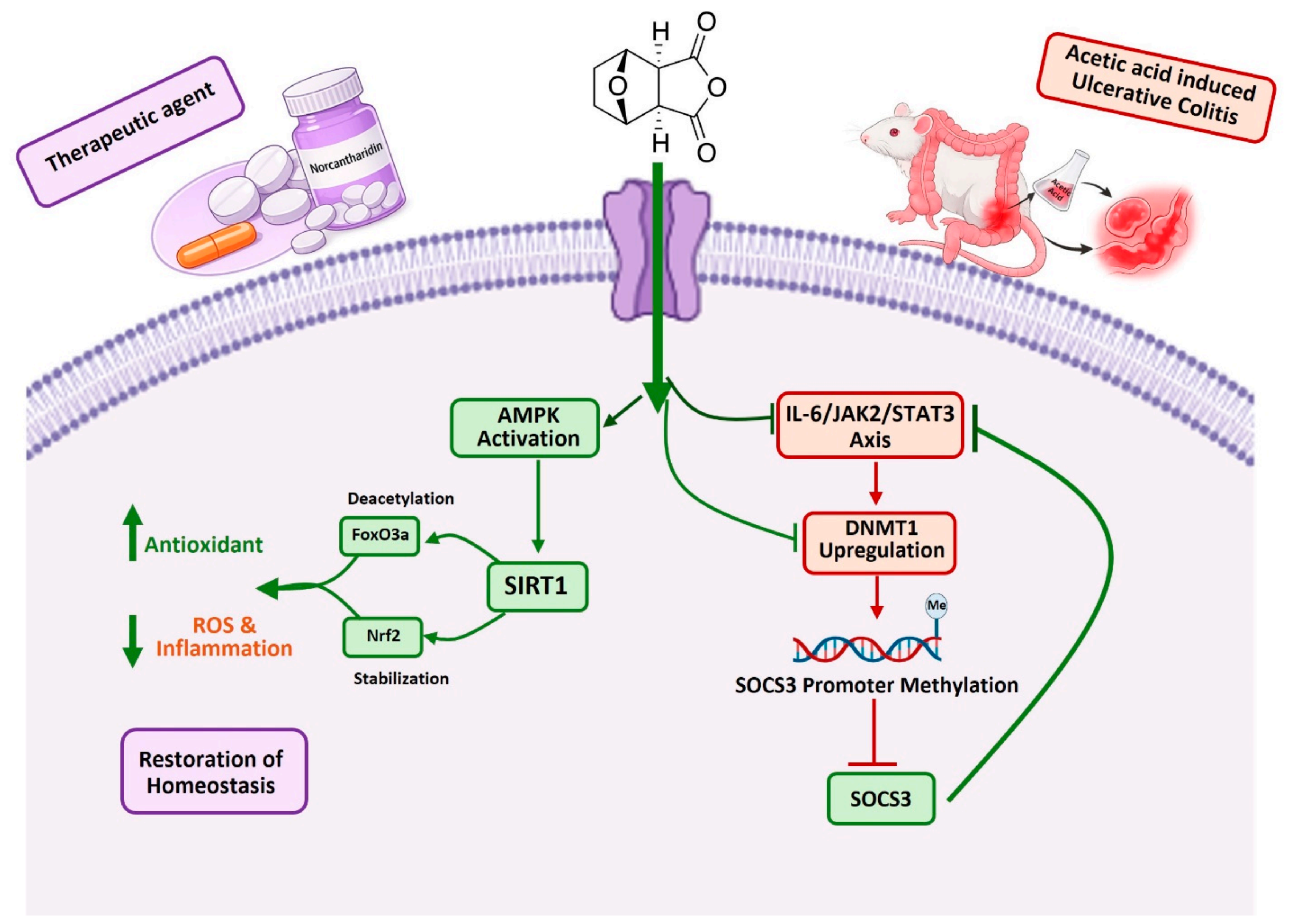
Therapeutic effects of norcantharidin, mesalazine, and their combination on ulcerative colitis through modulation of inflammatory, epigenetic, and metabolic signaling pathways. This figure illustrates the proposed mechanisms underlying the therapeutic effects of norcantharidin (NCTD) and mesalazine (5-ASA), alone or in combination, in the treatment of ulcerative colitis. The treatments are associated with modulation of key molecular pathways, including suppression of the IL-6/DNMT1/SOCS3 axis, which is associated with inflammation and epigenetic regulation, and activation of the AMPK/SIRT1/FOXO3a-Nrf2 pathway, which enhances antioxidant defense and cellular stress response. Collectively, these effects contribute to reduced inflammation, improved epithelial integrity, and attenuation of disease progression.

In conclusion, these findings suggest that NCTD, particularly in combination with 5-ASA, ameliorates experimental colitis and is associated with modulation of inflammatory, epigenetic, metabolic, and redox pathways. NCTD treatment was associated with alterations in IL-6/DNMT1/SOCS3 and AMPK/SIRT1/FOXO3a–Nrf2 signaling components together with reduced inflammation, oxidative stress, and indices of iron accumulation and lipid peroxidation (Figure 10). The combined NCTD/5-ASA regimen produced greater improvements in clinical, histopathological, and molecular parameters than either monotherapy. Collectively, these findings support a potential interaction between epigenetic and metabolic pathways in the regulation of inflammatory responses in experimental colitis. However, the observed molecular associations should be interpreted cautiously, and further mechanistic studies are required to establish causal relationships and evaluate the translational potential and long-term safety of this therapeutic approach.

## 4. Materials and Methods

### 4.1. Drugs and Chemicals

NCTD was purchased from Santa Cruz Biotechnology, Inc. (Dallas, TX, USA), while 5-ASA was obtained from Ferring Pharmaceuticals (Saint-Prex, Switzerland). Both agents were freshly suspended in 0.5% (*w*/*v*) carboxymethyl cellulose (CMC; El-Gomhouria Company, Cairo, Egypt). Acetic acid was purchased from Merck KGaA (Darmstadt, Germany) and prepared as a 4% (*v*/*v*) solution in normal saline for intracolonic instillation. Unless otherwise indicated, all other chemicals and reagents were obtained from Sigma-Aldrich (St. Louis, MO, USA), and all solvents were of analytical grade.

### 4.2. Animals

All animal experiments were performed in accordance with the National Institutes of Health Guide for the Care and Use of Laboratory Animals and were approved by the Research Ethics Committee of Horus University-Egypt, New Damietta, Egypt (Approval No. PH-2026-013; approval date: 18 February 2026). All efforts were made to minimize animal suffering and distress and to reduce the number of animals used. Thirty male Sprague-Dawley rats weighing 150–200 g were obtained from the animal house of VACSERA, Dokki, Giza, Egypt. Animals were housed in standard polypropylene cages, with three animals per cage, under controlled conditions of 25 ± 2 °C and a 12 h light/12 h dark cycle, with free access to a standard pellet diet (Meladco, El-Obour City, Cairo, Egypt) and water ad libitum. Rats were allowed to acclimatize for at least 1 week before the initiation of the experiment. Following acclimatization, animals were randomly assigned to five equal groups. The sample size was determined by an a priori statistical power analysis using G*Power version 3.1.9.4 (Heinrich-Heine-Universität Düsseldorf, Germany). The analysis was performed for a fixed-effects one-way ANOVA using an effect size (Cohen’s *f*) of 0.70, a significance level (α) of 0.05, a statistical power (1 − β) of 0.80, and five experimental groups. The calculated sample size supported the inclusion of six animals per group (30 animals in total). This sample size was also consistent with previous studies employing the same acetic acid-induced ulcerative colitis model [77]. No mortality or humane endpoints occurred during the study period; all animals were included in the final analysis.

### 4.3. Experimental Design and Colitis Induction

Following overnight fasting, rats were anesthetized with pentobarbital sodium (40 mg/kg, i.p.) [17]. The distal colon was gently irrigated with 1 mL of normal saline, and the lower abdomen was lightly palpated to evacuate residual fecal material. Experimental colitis was induced by slow intrarectal instillation of 1 mL of 4% (*v*/*v*) acetic acid in normal saline (Merck KGaA, Darmstadt, Germany) over 30 s using a polyurethane cannula (2 mm in diameter) inserted 6 cm through the anus. To prevent leakage, rats were maintained in a head-down position for 2 min after instillation. Control animals received an equivalent volume of intrarectal normal saline using the same procedure. This method was adopted in accordance with established AA-induced colitis protocols in rats [78].

Rats were randomly allocated into five groups (*n* = 6 per group) as follows:

- Control group: rats received 0.5% CMC orally once daily for 8 days.
- AA-colitis group: rats received 1 mL of 4% acetic acid intrarectally on day 0 and 0.5% CMC orally once daily for 8 days at the same volume used in the control group.
- NCTD group: rats received NCTD (10 mg/kg) orally suspended in 0.5% CMC once daily for 8 days [79], starting 24 h after AA induction.
- 5-ASA group: rats received 5-ASA (100 mg/kg) orally suspended in 0.5% CMC once daily for 8 days [80], starting 24 h after AA induction.
- NCTD/5-ASA group: rats received NCTD (10 mg/kg) and 5-ASA (100 mg/kg) orally, each suspended in 0.5% CMC, once daily for 8 days, starting 24 h after AA induction.

### 4.4. Assessment of Disease Activity Index (DAI)

Body weight, stool consistency, and rectal bleeding were recorded daily throughout the experimental period. The percentage of body weight loss was calculated relative to the initial body weight using the following equation:

Weight loss (%) = [(initial body weight − final body weight)/initial body weight] × 100

Fecal output was inspected visually each day to assess stool consistency and the presence of rectal bleeding. The disease activity index (DAI) was then calculated as the mean of the three clinical scores for body weight loss, stool consistency, and rectal bleeding, according to the following equation:

DAI = (body weight loss score + stool consistency score + rectal bleeding score)/3

Scoring of DAI parameters was performed as previously described [81,82] and is presented in Table 1. The DAI was used as an integrated indicator of colitis severity in the different experimental groups.

### 4.5. Colon Collection and Gross Morphometric Assessment

At the end of the experiment, rats were fasted overnight and, 24 h after the last treatment dose, were anesthetized with pentobarbital sodium (40 mg/kg, i.p.) and euthanized by exsanguination under anesthesia. The colon was carefully excised, rinsed with ice-cold phosphate-buffered saline, gently blotted dry, and stretched without tension. Colon length was measured from the distal rectum to the ileocecal junction, and colon weight was recorded. The distal 8 cm of the colon was divided longitudinally into two portions. One portion was immediately snap-frozen in liquid nitrogen and stored at −80 °C; this frozen tissue was subsequently subdivided into aliquots for ELISA (IL-6, TNF-α, SOCS3, SIRT1, HO-1), colorimetric assays (MDA, Fe^2+^), qRT-PCR (DNMT1), and Western blotting (p-AMPK). The other portion was fixed in 10% neutral buffered formalin (pH 7.4) for histopathological examination and immunohistochemistry (FOXO3a, Nrf2). All evaluations were performed on tissue derived from the same 8 cm distal segment per animal, ensuring regional consistency. To assess colonic edema and tissue swelling, the colon weight/length (W/L) ratio was calculated as follows [83]:

Colon W/L ratio (mg/cm) = colon weight (mg)/colon length (cm)

### 4.6. Histopathological Evaluation and Scoring of Colonic Injury

Colon tissues from the different experimental groups were fixed in 10% neutral buffered formalin for 24 h, washed with tap water, dehydrated through a graded series of ethanol, cleared in xylene, and embedded in paraffin. Paraffin-embedded tissues were sectioned at 4 µm, mounted on glass slides, deparaffinized, and stained with hematoxylin and eosin (H&E) for microscopic evaluation. Histopathological injury was assessed in the mid-colon region by a blinded histopathologist using a semiquantitative scoring system based on inflammatory cell infiltration, crypt damage, ulceration, and edema. The overall histopathological score was calculated as the sum of the individual component scores, yielding a maximum score of 13 (Table 2). All parameters were scored as whole integers; no partial or half-point scores were used. This scoring system was adapted from previously published methods for experimental colitis [84].

### 4.7. Molecular Docking

Molecular docking analysis was performed to investigate the binding interactions of NCTD with three selected protein targets: AMPK (PDB ID: 4CFE), SIRT1 (PDB ID: 5BTR), and DNMT1 (PDB ID: 7SFC). These targets were selected based on their established involvement in cellular energy metabolism, stress response, apoptosis regulation, and epigenetic modulation, which are mechanistically relevant to the reported pharmacological activities of NCTD. The three-dimensional crystal structures of the proteins were retrieved from the Protein Data Bank (PDB). Protein preparation was performed by removing water molecules and co-crystallized ligands, adding polar hydrogen atoms, assigning appropriate charges, correcting structural issues, and performing energy minimization to relieve steric clashes and stabilize receptor conformations. The ligand structure of NCTD was constructed and geometrically optimized prior to docking using standard energy minimization procedures. Molecular docking simulations were carried out using AutoDock Vina (version 1.1.2) [85]. For each protein, the docking grid box was centered on the co-crystallized ligand binding site. Grid box dimensions and coordinates were defined based on the position of the native ligand in the crystal structure. For each target, the docking grid box was centered on the co-crystallized ligand binding site. For each target, the docking grid box was individually centered on the corresponding co-crystallized ligand using target-specific center coordinates (x, y, and z) to accurately define the binding pocket. Although a uniform grid size of 20 × 20 × 20 Å was employed for all proteins, the grid center was adjusted for each receptor according to the position of its native ligand. The selected grid dimensions completely encompassed the respective binding pocket and surrounding key residues while providing sufficient space for ligand flexibility during docking. The docking calculations were performed using an exhaustiveness value of 20. Multiple binding poses were generated, and conformations were ranked according to the AutoDock Vina scoring function. The binding pose with the lowest predicted binding free energy (kcal/mol) was selected for further interaction analysis and visualization. To validate the docking protocol, the co-crystallized ligand of each target was extracted and redocked into the corresponding binding site using the same docking parameters. The validation yielded RMSD values of 1.749 Å (AMPK), 0.534 Å (SIRT1), and 1.605 Å (DNMT1), confirming that the docking protocol reliably reproduced the experimental binding poses (RMSD < 2.0 Å). The resulting ligand–protein interactions, including hydrogen bonding, hydrophobic interactions, and π-related contacts, were analyzed and visualized using Discovery Studio Visualizer (Dassault Systèmes, San Diego, CA, USA) [86], enabling the generation of both two-dimensional and three-dimensional interaction representations.

### 4.8. Biochemical and Immunoassay Analyses of Colon Tissues

Colon tissue homogenates obtained from the different experimental groups were used for biochemical and immunoassay analyses. Colonic malondialdehyde (MDA) levels (Cat. No. MD 25 28, Bio-Diagnostic, Dokki, Giza, Egypt) and ferrous iron (Fe^2+^) levels (Cat. No. K390-100, BioVision, Milpitas, CA, USA) were measured colorimetrically according to the manufacturers’ instructions. In parallel, colonic levels of IL-6 (Cat. No. PR6000B, R&D Systems, Minneapolis, MN, USA), tumor necrosis factor-α (TNF-α) (Cat. No. CSB-E11987r, Cusabio, Wuhan, Hubei, China), suppressor of cytokine signaling 3 (SOCS3) (Cat. No. ELK6764, ELK Biotechnology, Wuhan, Hubei, China), SIRT1 (Cat. No. MBS2600246, MyBioSource, San Diego, CA, USA), and heme oxygenase-1 (HO-1) (Cat. No. MBS764989, MyBioSource, San Diego, CA, USA) were quantified using commercially available rat-specific ELISA kits in accordance with the manufacturers’ instructions.

### 4.9. Quantitative Real-Time PCR Analysis of Colonic DNMT1 mRNA Expression

Colonic DNA methyltransferase 1 (DNMT1) mRNA expression was quantified by quantitative real-time polymerase chain reaction (qRT-PCR). Total RNA was isolated from colon tissue using the RNeasy Mini Kit (Qiagen, Hilden, Germany) in accordance with the manufacturer’s instructions. RNA purity was assessed spectrophotometrically using the A260/A280 ratio. For cDNA synthesis, 2 μg of total RNA was reverse-transcribed using the SensiFAST™ cDNA Synthesis Kit (Bioline, Meridian Bioscience, Memphis, TN, USA). qRT-PCR was performed on a StepOne™ Real-Time PCR System (Applied Biosystems, Thermo Fisher Scientific, Waltham, MA, USA) in a final reaction volume of 20 μL containing 10 μL TOPreal™ SYBR Green qPCR 2× PreMIX (Enzynomics, Daejeon, Republic of Korea), 2 μL cDNA template, 1 μL each of forward and reverse primers (10 μM stock concentration; final concentration, 0.5 μM each), and 6 μL nuclease-free water. Primers were synthesized by Invitrogen (Thermo Fisher Scientific, Waltham, MA, USA), and their sequences are listed in Table 3. β-actin served as the endogenous reference gene, and relative DNMT1 mRNA expression was determined using the 2^−ΔΔCt^ method.

### 4.10. Immunohistochemical Assessment of Colonic FOXO3a and Nrf2 Expression

5 μm paraffin-embedded colon sections were incubated overnight at 4 °C with rabbit polyclonal primary antibodies against FOXO3a (Cat. No. GTX100277, GeneTex, Irvine, CA, USA; 1:500) and nuclear factor erythroid 2-related factor 2 (Nrf2) (Cat. No. GTX55732, GeneTex, Irvine, CA, USA; 1:200). Sections were then incubated with horseradish peroxidase-conjugated goat anti-rabbit IgG H&L secondary antibody (Cat. No. ab6721, Abcam, Cambridge, UK), and immunoreactivity was visualized with 2% diaminobenzidine prepared in 50 mM Tris buffer (pH 7.6). Slides were examined and imaged using a light microscope (Olympus CH2, Olympus, Tokyo, Japan). Immuno-positive areas were quantified using ImageJ software (version 1.53c; National Institutes of Health, Bethesda, MD, USA) in three fields per section. Fields were selected via systematic random sampling at ×100 magnification, avoiding tissue edges, large artifact regions, and areas of folding or tearing. Image capture and quantification were performed by an assessor blinded to treatment group allocation. Results were expressed as the percentage of positive staining area. Representative images were captured at original magnifications of ×100 and ×400, with scale bars of 100 μm and 50 μm, respectively.

### 4.11. Western Blot Analysis of Colonic p-AMPK Protein Expression

Protein expression of phospho-AMPKα1 (Thr172) in colon tissue samples was evaluated by Western blotting. Colon tissues were homogenized in ice-cold RIPA buffer (Thermo Fisher Scientific, Cat. No. 89900, Waltham, MA, USA) supplemented with protease and phosphatase inhibitors to extract total protein. Protein concentration was determined using a BCA protein assay kit (Thermo Fisher Scientific, Cat. No. 23225, Waltham, MA, USA). Equal amounts of protein were mixed with Laemmli sample buffer (Bio-Rad, Cat. No. 1610747, Hercules, CA, USA) and heated at 95 °C for 5 min. Proteins were separated on 8% SDS-PAGE gels along with molecular weight markers (Bio-Rad, Cat. No. 4561094, Hercules, CA, USA) and then transferred onto PVDF membranes (Millipore, Cat. No. IPVH00010, Burlington, MA, USA). Membranes were blocked with 5% non-fat dry milk in TBST (Tris-buffered saline containing 0.05% Tween-20) for 1 h 30 min at room temperature, followed by overnight incubation at 4 °C with primary antibodies against p-AMPK (Thr172) (rabbit polyclonal, Cat. No. PA5-17831, Thermo Fisher Scientific, Waltham, MA, USA; 1:500) and β-actin (mouse monoclonal, clone 15G5A11/E2, Cat. No. MA1-140, Thermo Fisher Scientific, Waltham, MA, USA; 1:5000) as a loading control. After washing, membranes were incubated with the appropriate horseradish peroxidase-conjugated secondary antibodies (1:5000) for 1 h at room temperature. Protein bands were visualized using a chemiluminescent detection system and imaged with a ChemiDoc MP Imaging System (Bio-Rad, Hercules, CA, USA). Band intensities were quantified densitometrically and normalized to β-actin.

### 4.12. Statistical Analysis

Statistical analysis was performed using GraphPad Prism version 10.4.1 (GraphPad Software, San Diego, CA, USA). Data were first assessed for normality using the Shapiro–Wilk test. Normally distributed data were expressed as mean ± SE and analyzed using one-way ANOVA followed by the Tukey–Kramer multiple-comparisons post hoc test, which was used to account for multiple pairwise comparisons while controlling the family-wise error rate. The total histopathological score was analyzed using the Kruskal–Wallis test followed by Dunn’s multiple-comparisons test. A two-tailed *p* value < 0.05 was considered statistically significant.

## Figures and Tables

**Table 1:** Disease activity index (DAI) scoring criteria for assessment of colitis severity in rats.

Score	Body Weight Loss (%)	Stool Consistency	Rectal Bleeding
0	None	Normal	Negative
1	1–5	—	—
2	6–10	Loose stools	Hemoccult positive
3	11–20	—	—
4	>20	Watery diarrhea	Gross bleeding

Normal stools = well-formed pellets; loose stools = pasty stools that do not adhere to the anus; watery diarrhea = liquid stools that adhere to the anus.

**Table 2:** Histopathological scoring system for evaluation of colonic mucosal injury.

Parameter	Score	Criteria
Inflammatory cell infiltration	0	None
	1	Occasional inflammatory cells limited to the submucosa
	2	Significant focal inflammatory cell infiltration in the submucosa
	3	Significant focal inflammatory cell infiltration in the submucosa and lamina propria
	4	Extensive inflammatory cell infiltration in the submucosa, around blood vessels, and lamina propria
	5	Transmural inflammatory cell infiltration extending from the mucosa to the muscularis
Crypt damage	0	No change
	1	Mild crypt damage with increased spaces between crypts
	2	Goblet cell loss and shortening of some crypts
	3	Large areas without crypts
	4	Complete loss of crypts
Ulceration	0	None
	1	Small focal ulcer
	2	Frequent small ulcers
	3	Large ulcerated areas with loss of surface epithelium
Edema	0	Absent
	1	Present

Total histopathological score = sum of all parameter scores; maximum score = 13.

**Table 3:** Primer sequences used for quantitative real-time PCR.

Gene	Description	Primer Sequence	Reference Sequence	Product Size
DNMT1	F	5′-CAGCTACCTCGGTGGGTTAC-3′	NM_053354.3	199
	R	5′-CGACATCACGATCCCAGGAC-3′		
β-actin	F	5′-CTGTGTGGATTGGTGGCTCT -3′	NM_031144.3	134
	R	5′-AGCTCAGTAACAGTCCGCC-3′		

DNMT1, DNA methyltransferase 1; F, forward; R, reverse.

## Data Availability

The data that support the findings of this study are available in the Materials and Methods, and Results of this article.

## Supplementary Materials

The following supporting information can be downloaded at: https://www.mdpi.com/article/10.3390/ijms27156666/s1.

## Abbreviations

5-ASA	Mesalazine
AA	Acetic acid
AMPK	Adenosine monophosphate-activated protein kinase
DAI	Disease activity index
DNMT1	DNA methyltransferase 1
H&E	Hematoxylin and eosin
HO-1	Heme oxygenase-1
IBD	Inflammatory bowel disease
IL-6	Interleukin-6
NCTD	Norcantharidin
Nrf2	Nuclear factor erythroid 2-related factor 2
qRT-PCR	Quantitative real-time polymerase chain reaction
SIRT1	Sirtuin-1
SOCS3	Suppressor of cytokine signaling 3
TNF-α	Tumor necrosis factor-α
UC	Ulcerative colitis
